# Chronic stress and adipose tissue in the anorexic state: endocrine and epigenetic mechanisms

**DOI:** 10.1080/21623945.2020.1803643

**Published:** 2020-08-10

**Authors:** Yang Xiao, Dongmin Liu, Mark A. Cline, Elizabeth R. Gilbert

**Affiliations:** a Department of Animal and Poultry Sciences, Virginia Polytechnic Institute and State University, Blacksburg, VA, USA; b Department of Human Nutrition, Foods, and Exercise, Virginia Polytechnic Institute and State University, Blacksburg, VA, USA; c School of Neuroscience, Virginia Polytechnic Institute and State University, Blacksburg, VA, USA

**Keywords:** Adipocyte, adipose tissue, anorexia, stress, epigenetics, endocrine

## Abstract

Although adipose tissue metabolism in obesity has been widely studied, there is limited research on the anorexic state, where the endocrine system is disrupted by reduced adipose tissue mass and there are depot-specific changes in adipocyte type and function. Stress exposure at different stages of life can alter the balance between energy intake and expenditure and thereby contribute to the pathogenesis of anorexia nervosa. This review integrates information from human clinical trials to describe endocrine, genetic and epigenetic aspects of adipose tissue physiology in the anorexic condition. Changes in the hypothalamus-pituitary-thyroid, -adrenal, and -gonadal axes and their relationships to appetite regulation and adipocyte function are discussed. Because of the role of stress in triggering or magnifying anorexia, and the dynamic but also persistent nature of environmentally-induced epigenetic modifications, epigenetics is likely the link between stress and long-term changes in the endocrine system that disrupt homoeostatic food intake and adipose tissue metabolism. Herein, we focus on the adipocyte and changes in its function, including alterations reinforced by endocrine disturbance and dysfunctional adipokine regulation. This information is critical because of the poor understanding of anorexic pathophysiology, due to the lack of suitable research models, and the complexity of genetic and environmental interactions.

## Introduction

There are two major types of adipose tissue – brown adipose tissue (BAT), responsible for non-shivering thermogenesis, and white adipose tissue (WAT), which is the primary site for energy storage [1]. Adipose tissue is one of the largest endocrine organs, secreting a diverse array of hormones and cytokines (i.e. adipokines) that regulate physiological changes such as energy intake and metabolism, thermoregulation, and glucose homoeostasis [2–4].

Obesity is associated with abnormal adipose tissue metabolism and function. Likewise, a lack of adipose tissue is associated with metabolic dysregulation. For instance, in lipodystrophy syndrome – a genetic or acquired disorder characterized by a lack of adipose tissue, patients have disproportionate hyperphagia, reproductive dysfunction, dyslipidemia, and insulin resistance, etc [5]. In anorexia nervosa (AN) – an eating disorder characterized by reduced food intake, the loss of body weight and fat mass [6] can also disturb the endocrine system and lead to the sequential physiological processes that exacerbate the condition [7]. Compared to healthy counterparts, patients with AN have elevated salivary, plasma and urinary cortisol concentrations [8], which are associated with greater levels of stress, anxiety and depression [9]. In turn, exposure to stress is associated with the pathogenesis of AN [10–12] and obesity [13] through the interruption of energy homoeostasis.

This review focuses on the relationship between chronic stress and adipose tissue physiology in the anorexic state. We discuss genetic and epigenetic aspects of metabolic changes in adipose tissue and associated mechanisms, summarizing knowledge from human clinical trials and some animal models, with a focus on stress-induced epigenetic modifications that affect the endocrine system and adipose tissue physiology. Such information provides new insights for the direction of research in adipose tissue metabolism in the anorexic state.

## Fat mass and adipose tissue distribution

According to the Diagnostic and Statistical Manual of Mental Disorders, anorexia nervosa is defined as a restricted energy intake relative to the requirement due to the intense fear of weight gain even at a significantly low weight, where the body mass index (BMI) is below 18 in adults and the corresponding BMI percentile in children and adolescents [14]. In the United States, the prevalence of AN is 0.6 ± 0.2% in adults, with a significant difference between females (0.9 ± 0.3%) and males (0.3 ± 0.1%) [15]. Similar patterns are observed in the UK and the Netherlands, where the incidence rate in females is higher than in males, with the peak incidence rate in adolescent girls [16,17]. A meta-analysis of 62 studies published from 1996 to 2019 showed that female AN patients had 50% less body fat mass than their healthy counterparts on average, with fat being stored mainly in the trunk region [18]. Body fat loss has been observed in different age groups. Adolescent girls with AN had about 1/3 of the total body fat mass (4.1 ± 2.8 kg vs. 12.1 ± 2.9 kg) and half of the body fat mass as a percentage of body weight (10.1 ± 5.9% vs. 23.1 ± 3.8%) compared to age-matched healthy controls [19]. In adult women with AN, total body fat mass (4.0 ± 3.0 kg) and percentage fat mass (9.8 ± 6.6%) were also lower than their age-matched healthy controls (11.8 ± 1.8 kg and 23.0 ± 3.6%, respectively) [19]. Interestingly, both adolescent girls and adult women with AN had lower percentages of extremity fat (adolescent 50.0 ± 6.7% vs. 57.4 ± 3.3%; adult 48.8 ± 10.2% vs. 57.8 ± 4.5%) but higher percentages of trunk fat (adolescent 40.5 ± 5.5% vs. 35.6 ± 3.4%; adult 41.8 ± 8.5% vs. 35.4 ± 4.5%) compared to their respective healthy counterparts, indicative of fat redistribution with a preferred loss of extremity fat under the AN state in females [19]. However, the higher percentage of trunk fat observed here is contradictory to previous reports that the percentage of trunk fat was lower in adolescent girls [20] and not different in adult women [21] from their respective age-matched healthy controls. It should be noted that subjects recruited in these studies represented a range of ages and duration of AN, as well as menarche and menstruation status, which may all contribute to differences in fat redistribution. Similar to the observations in females, adolescent boys with AN had less fat mass (6.7 ± 0.5 kg), percentage fat mass (12.8 ± 0.8%) and percentage extremity fat (49.9 ± 1.6% of total fat mass) as compared with age-matched healthy controls (9.7 ± 0.7 kg, 15.8 ± 1.1% and 55.5 ± 0.9%, respectively), while the percentage of trunk fat was comparable to the controls (35.4 ± 1.2% vs. 33.5 ± 0.9%) [22]. In adult men, percentage trunk fat was higher in AN than age-matched healthy subjects (48.8 ± 4.0% vs. 44.3 ± 4.8%) while percentage leg fat was comparable between the two groups [23]. Other studies on body fat distribution in the AN state, ranging from 1989 to 2014, were comprehensively reviewed in [24], including 7 studies that were conducted in adolescents (1 on male and 6 on female subjects), and 13 that focused on adult AN (all on female subjects). The main findings from the review were that: 1) adolescent girls with AN experienced a significant loss of trunk fat while their loss of extremity fat as a percentage of body weight varied; 2) adolescent boys with AN, in contrast to the girls, had a higher percentage of trunk fat than healthy controls but a preferential loss of fat in the extremities, which was hypothesized to be related to lower testosterone levels; 3) adult females with AN had similar waist to hip or android (trunk and upper body) to gynoid (around the hips, breasts and thighs) ratios as healthy controls, but tended to lose more extremity fat than trunk fat in the AN state.

There are also BAT differences in AN patients. One study reported that adult female AN patients displayed no detectable BAT activity under 22–24 °C before or after weight regain but a lower resting metabolic rate compared to the healthy counterparts [25]. Under cold exposure, only 1 out of 5 female AN patients and 2 out of weight-regained AN patients showed BAT activity, while BAT activity was detectable in 4 out of 5 healthy controls [26]. These results collectively suggest that there is an adaptive reduction in resting metabolic rate in AN patients to compensate for chronic fuel deficiency caused by restrictive eating behaviour.

## Depot-specific adipose tissue physiology

While substantial loss of adipose tissue in the trunk and extremity regions was reported in AN patients across different age groups, only a few studies distinguished between the loss of subcutaneous (sWAT) versus visceral adipose tissue (vWAT) in the trunk region. In adult female, AN subjects, the loss of vWAT contributed to less than 20%, while sWAT contributed to over 80% of the total loss of abdominal adipose tissue [27,28]. Consistent with these results, a study of 14 adult female AN patients showed that the cross-sectional area of vWAT was about 54% of the healthy controls while that of sWAT was only about 49% [29], indicating a greater loss of sWAT than vWAT in the AN state, similar to the observations reviewed in [24]. These studies also reported an average of about 50% loss of thigh fat in the female AN subjects. Adipocyte area within the abdominal sWAT of adult female AN subjects was smaller than in healthy counterparts [30]. Fat attenuation – an indirect measurement of tissue quality and composition, was also higher in abdominal sWAT, vWAT, as well as thigh fat and intramuscular AT (iMAT) of adult AN women compared to age-matched healthy controls, reflective of smaller adipocytes with reduced lipid content in these depots, which is a marker of systemic fibrosis [31].

Although research on abdominal and thigh fat in AN is limited, there is considerable focus on the marrow adipose tissue (MAT). In contrast to the reduced abdominal sWAT and vWAT as well as thigh fat and iMAT, there was increased MAT in the lumbar [27,32,33], femur [27] and tibia [32] in adolescent girls [32,34] and adult women [27,29], as well as in the iliac crest of an adult male [35] with AN. There was an inverse correlation between MAT/fat content in the lumbar and femur with body mass index (BMI) and thigh fat mass, and femur MAT was also inversely correlated with abdominal total adipose tissue and sWAT masses [27]. These data collectively suggest that adipose tissue is re-distributed in the AN state, with the most potent catabolism from sWAT accompanied by increased deposition of MAT.

## Endocrine disruption in AN

Serving as a key endocrine organ, WAT secretes over 700 confirmed and putative proteins [36], including a myriad of hormones (e.g. leptin, adiponectin, resistin and glucocorticoids) and pro-inflammatory factors (e.g. tumour necrosis factor α [TNFα] and interleukin 6 [IL-6]). In addition, WAT cells contain receptors for insulin, leptin, steroid hormones (e.g. glucocorticoids, oestrogen and androgen), thyroid hormone, and catecholamines, etc. (reviewed in [3]). Thus, WAT is an important regulator of diverse physiological processes that ultimately impact energy balance and itself is an important target of other organs that contribute to maintaining energy homoeostasis through regulating its storage and expenditure of energy in the form of fatty acids.

**Figure 1.**
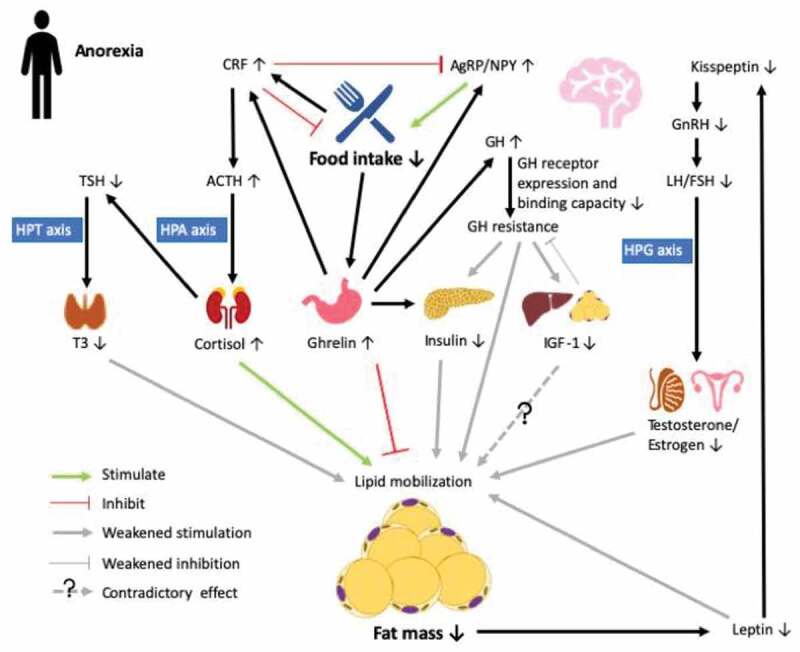
Endocrine abnormalities in the anorexic state (AN). Decreased food intake in anorexia stimulates the secretin of ghrelin in the stomach, which promotes food intake by upregulating agouti-related peptide/neuropeptide Y (AgRP/NPY) neurons in the arcuate nucleus (ARC) of the hypothalamus. Ghrelin acts on the somatotrophs in the pituitary to stimulate the release of growth hormone (GH) to maintain balance in lipid deposition and mobilization, meanwhile activating corticotropin-releasing factor (CRF) neurons in the paraventricular nucleus, which counteracts effects on AgRP/NPY and inhibits food intake. In AN, increased GH secretion is accompanied by lowered GH receptor expression and binding capacity in the liver, leading to GH resistance and further reduced effects on inhibiting lipid mobilization and stimulating insulin-like factor 1 (IGF-1) expression. In turn, IGF-1 provides weak negative feedback to GH production, which further promotes GH resistance. Ghrelin also inhibits insulin secretion by modulating ATP production so as to maintain euglycemia. Chronic fasting is a stressor that directly upregulates the expression of CRF to activate the hypothalamic-pituitary-adrenal (HPA) axis. Elevated cortisol sequentially suppresses the release of thyroid-stimulating hormone (TSH), which then reduces the activation of triiodothyronine (T3) via the hypothalamic-pituitary-thyroid (HPT) axis to reduce energy expenditure. Loss of fat mass under AN also reduces the production of adipokine leptin, which further downregulates the expression of kisspeptin in the ARC. Reduced kisspeptin then leads to reduced secretion of sex hormones through regulation of the hypothalamic-pituitary-gonadal (HPG) axis, which eventually weakens lipid mobilization. Therefore, in AN, prolonged negative energy balance coupled to endocrine disruption may result in reduced lipid mobilization to preserve energy for vital maintenance

Adipose tissue function is directly impacted by changes in appetite regulation. In AN patients, food intake is substantially reduced, which triggers the release of ghrelin – an orexigenic peptide secreted from the hypothalamic arcuate nucleus (ARC) and stomach. Ghrelin acts on the growth hormone secretagogue receptor 1a in the hypothalamus and triggers the production of orexigenic peptides agouti-related peptide (AgRP) and neuropeptide Y (NPY) in the ARC to promote food intake [37] (Figure 1). Meanwhile, ghrelin also acts on somatotrophs in the pituitary to stimulate the release of growth hormone (GH) [38]. While ghrelin promotes lipogenesis and inhibits lipolysis [39], GH inhibits lipid accumulation and potentiates lipid mobilization [40]. GH sequentially increases the production of insulin-like growth factor 1 (IGF-1) – a factor highly expressed in liver and adipose tissue [40], whereas its function in adipogenesis is controversial [40]. Ghrelin also plays a critical role in maintaining glucose homoeostasis in the fasting state. Uncoupling protein 2 (UCP2), which decreases ATP production and thus the ATP to ADP ratio, is upregulated by ghrelin to inhibit the secretion of insulin [41]. On the other hand, ghrelin increases hepatic glucose output by stimulating gluconeogenesis through the activation of the GH pathway [41]. Elevated ghrelin and GH levels, together with hypoinsulinemia, were observed in adult female AN patients. However, hepatic GH receptor expression and binding capacity were low, leading to GH resistance and consequently, reduced expression of IGF-1 [42], which is also observed in adolescent girls [20]. In turn, reduced IGF-1 provides weak negative feedback on GH secretion, which may help maintain the high level of GH expression, and thus potentiates lipolytic activity to provide substrates for gluconeogenesis and maintaining euglycemia [7]. Surprisingly, weight recovery in AN patients was associated with less insulin-dependent glucose uptake than in both healthy and no weight recovery controls, which is indicative of decreased insulin sensitivity [43]. This is postulated to be attributable to the loss of sWAT during the early stage of AN, which may impair lipid storage and restoration capabilities in the depot and thus divert lipid storage mainly to vWAT or other tissues (e.g. muscle and liver), eventually leading to insulin resistance [43].

While changes in energy intake directly affect the function and mass of adipose tissue, in turn, changes in adipocyte abundance and consequently alterations in adipokine secretion, reciprocally affect appetite regulation, and directly impact other pathways that affect glucose homoeostasis and reproductive function. Leptin is an adipose-derived cytokine that is secreted in proportion to body fat mass. Like ghrelin, leptin is also a major regulator of appetite via receptor interactions in the ARC [3]. With the reduction in body fat mass in the AN state, serum/plasma leptin levels decreased in both adolescent girls [20] and adult women [30,44]. Although not specifically studied in male AN patients, 72 hr of fasting in healthy lean men led to reduced serum leptin and IGF-1 concentrations, whereas daily administration of replacement doses of leptin restored IGF-1 levels to those on the initial day of the experiment [45], demonstrating the interaction between leptin and IGF-1 during energy balance regulation. Indeed, leptin and IGF-1 expression in adipose tissue are positively correlated whereas an antagonistic effect between leptin and IGF-1 in adipocytes was also observed [40], and GH secretion in fasted rats was stimulated by leptin administration [46].

In addition to modulating the activity of hypothalamic appetite-regulatory neurons, leptin regulates kisspeptin neurons in the ARC [47]. Kisspeptin is involved in the regulation of pulsatile gonadotropin-releasing hormone (GnRH) secretion, which sequentially stimulates the pituitary release of luteinizing hormone (LH) and follicle-stimulating hormone (FSH) to initiate puberty or regulate ovulation through the promotion of sex steroid hormone (i.e. testosterone in men and oestrogens in women) secretion [48], also known as the hypothalamus-pituitary-gonadal (HPG) axis. Thus, it could be speculated from the human studies that leptin and kisspeptin act in concert to affect puberty and reproduction, and lower leptin contributes to the reduced kisspeptin levels and GnRH secretion, leading to lower testosterone and oestrogen secretion in fasting males and AN females, respectively [49,50].

Testosterone enhances protein kinase A-mediated adipocyte lipolysis through upregulating the expression of β-adrenergic receptors [51]. Meanwhile, testosterone inhibits lipid uptake via downregulating lipoprotein lipase (LPL) activity [52]. Consistently, testosterone administration reduced vWAT accumulation in men [53] as well as adipocyte size in hypogonadal male rats [54]. Therefore, decreased secretion of testosterone may be associated with the observed maintenance of percentage trunk fat while reducing extremity fat in adolescent boys with AN [22]. Oestrogen was also inversely associated with fat attenuation in both sWAT and vWAT in AN females [31]. Chronically reduced synthesis of oestrogen causes amenorrhoea in female AN patients, and resumption of menses is considered to be a biological indicator of health status after weight restoration, which is associated with a higher percentage of body fat mass [50]. Interestingly, plasma kisspeptin is positively correlated with BMI and body fat mass in premenopausal adult females with AN [49] while inversely correlated with BMI in adolescent girls [55], indicating different regulatory patterns of HPG axis activation in adolescent and adult females in the AN state. Although leptin mRNA in abdominal sWAT was decreased in AN patients [56], free leptin concentrations in abdominal sWAT were similar to those in the healthy individuals [30]. In contrast, increased mRNA expression of resistin, an adipocyte-derived hormone that is highly expressed in the abdominal fat and is involved in insulin resistance and energy balance, was associated with greater amounts of the hormone in abdominal sWAT [44], despite that circulating resistin was either unchanged [56] or decreased [44] in AN patients compared to healthy counterparts.

There is also a direct effect of the stress response on the regulation of appetite and adipose tissue physiology. Chronic starvation activates the stress cascade, also known as the hypothalamus-pituitary-adrenal (HPA) axis, to further interfere with the hormonal regulation of ARC neuronal and adipocyte functions. One critical factor responsible for stress cascade activation is corticotropin-releasing factor (CRF), also referred to as corticotropin-releasing hormone. Upon starvation, increased CRF expression in the hypothalamic paraventricular nucleus (PVN) promotes the release of adrenocorticotropic hormone (ACTH) from the anterior pituitary [57]. ACTH then targets the adrenal cortex to stimulate the production of cortisol, which in adipocytes facilitates lipid accumulation in the presence of insulin while promoting lipid mobilization in the presence of GH [58]. In adult female AN patients, urinary-free cortisol was positively correlated with thigh fat while its correlation with cross sectional area in abdominal sWAT and vWAT was negative [31]. Moreover, release of CRF onto the ARC suppresses appetite and promotes energy expenditure by inhibiting NPY/AgRP neuronal activity [59]. Ghrelin can activate CRF neurons in the PVN independent of the mediation of ARC AgRP/NPY neurons, which provides another mechanism for elevating cortisol levels in the fasting state [60].

Elevated cortisol suppresses the release of thyroid-stimulating hormone (TSH) from the anterior pituitary, which is also inhibited by ghrelin [61]. TSH, the release of which is normally stimulated by thyroid releasing hormone from the hypothalamus, sequentially activates triiodothyronine (T3) [62], thus stress can decrease activity of the hypothalamus-pituitary-thyroid (HPT) axis. Hence, lowered T3 was observed together with low BMI and leptin levels as well as increased ghrelin and cortisol, in the AN state [7,20,63]. T3 plays an important role in regulating energy balance. Reduced circulating T3 results in decreased resting energy expenditure and adipocyte lipolytic activities [64], which is pivotal in the conservation of energy for vital functions in the AN or fasting state. Cortisol, ghrelin [65] and GH [66] are positively correlated, while leptin [67], IGF-1 [65], and testosterone and oestrogen [68] as well as T3 [69] are negatively correlated with MAT mass. Hence, increased cortisol, ghrelin, and GH, accompanied by reduced leptin, IGF-1, sex steroid hormones, and T3 in AN, are also associated with suboptimal bone health.

It should be noted that there is limited knowledge on the regulation of lipolysis in AN. Although lipolysis can contribute to the energy requirement for maintenance under a negative energy balance state, in adult female AN patients, fasting plasma glycerol [70,71] or free fatty acids (FFA) [72] did not differ from age-matched healthy controls, whereas glycerol concentrations in sWAT were indeed greater in the AN state [70]. In adolescent girls with AN, fasting plasma glycerol as well as glycerol production varied considerably among patients. Although glycerol production rate was inversely correlated with body weight, suggesting that there is more lipolytic activity in those with lower body weight, no such correlation was observed between plasma glycerol concentration and body weight or BMI [73]. Since the duration or severity of AN in each patient was not specified, it is not known whether such differences contributed to the variation among patients. It is possible that during the early stage of AN, lipolysis increases to facilitate a return to homoeostasis, whereas under prolonged fasting-induced negative energy balance, lipolysis decreases to preserve energy required for vital maintenance. This idea is supported by the observations that adult female AN patients had lower heart rates and blood pressures than their healthy counterparts [70,71]. A recent study revealed that *NPY*-knockout mice displayed greater rates of lipolysis, fat mass loss and mortality during prolonged calorie restriction than wild-type healthy controls [74], suggesting that altered gene expression among patients may also contribute to the variations in lipolysis.

In summary, although cause and effect relationships between stressors, reduced appetite, and metabolic disruptions in the anorexic state are unclear, there are obvious changes in major hormonal systems that exert effects on both appetite regulation and lipid mobilization in adipose tissue that eventually negatively impact other important physiological functions such as reproduction. In general, there is reduced activity of the HPT and HPG axes, coupled to increased activity of the HPA cascade, that interact to intensify hypothalamic anorexigenic pathways, a condition that is further exacerbated by reduced fat mass and dysregulation of adipocyte lipid metabolism and adipokine secretion.

## Genetic association of AN and lipid metabolism

Based on family and twin studies, the symptoms/behaviours of AN are heritable, where genetic heritability explains about 46–72% of the eating behaviour (restrictive and binge-purging/vomiting) and 32–72% of the pathological attitudes (e.g. body dissatisfaction, weight concern and weight preoccupation), with higher heritability observed in post- than pre-pubertal twins [75]. Moreover, familial co-transmission of psychological (major depression and anxiety) and personality traits (reactivity towards stress, negative emotionality and harm avoidance) both contributed to the pathogenesis of AN [75]. The estimated overall heritability of AN is 0.56 based on the large cohort twin study, with the rest determined by environmental factors [76], while the partitioning single-nucleotide polymorphism (SNP) heritability of AN is 0.20, indicating the contribution of genetic variation to twin-based heritability of AN [77]. As reviewed in [78] and [79], SNPs in the genes encoding serotonin receptors, serotonin transporter, dopamine receptors DRD2 and DRD4, catechol-O-methyltransferase (COMT), leptin and leptin receptor, ghrelin, melanocortin 4 receptor (MC4R), proopiomelanocortin (POMC), AgRP, oestrogen receptors ESR1 and ESR2/ESβ, brain-derived neurotrophic factor (BDNF), cannabinoid receptor 1 (CNR1), opioid receptor delta 1 (OPRD1), as well as fat mass- and obesity-associated gene (FTO) are associated with the risk of AN. Among these, serotonin receptors, serotonin transporter, dopamine receptors, COMT, ghrelin, MC4R, POMC, AgRP, BDNF, CNR1 and OPRD1 indirectly affect adipose tissue metabolism through hypothalamic appetite/body weight regulation, whereas leptin and leptin receptor, oestrogen receptors and FTO [80] can directly act on the adipocyte to regulate lipid metabolism.

A recent genome-wide association study (GWAS) revealed one locus with genome-wide significance for a single variant on chromosome 12, which covers 6 genes including zinc finger CCCH-type containing 10 (*ZC3H10*) and erb-b2 receptor tyrosine kinase *ERBB3* [77]. ZC3H10 is an activator of UCP1 and promotes brown adipocyte differentiation, and transgenic overexpression of ZC3H10 prohibits diet-induced obesity [81], while the expression of ERBB3 was downregulated during lipogenesis in sebocytes [82]. Additionally, there was a positive genetic correlation between AN and high-density lipoprotein (HDL) cholesterol, and negative correlation between AN and BMI, insulin, glucose, low-density lipoprotein cholesterol (LDL) and very low-density lipoprotein cholesterol [77].

Gene-tissue associations from a transcriptomic imputation study attributed 13.9% of the phenotype variance in AN to sWAT [83]. Moreover, decreased expression of receptor accessory protein 5 in the dorsolateral prefrontal cortex, which promotes the expression of olfactory receptors and is positively correlated with body weight, is associated with a higher risk of AN [83], probably due to the dysfunction of odour discrimination and sequential desire of food.

Transcriptomic analysis on cortical neurons differentiated from induced pluripotent stem cells revealed higher expression of the TSH receptor and diacylglycerol kinase gamma (*DGKG*) in AN than in healthy females [84]. As a component of the HPT axis, TSH is involved in central regulation of energy homoeostasis [85]. In addition to the thyroid, the TSH receptor is also found in preadipocytes and mature adipocytes, and its activation potentiates adipogenesis in mouse 3T3-L1 (preadipocyte) cells, while inhibiting expression of fatty acid synthase, an enzyme important for lipogenesis in the adipose tissue of rodents [86]. *DGKG* expression increases in the prefrontal cortex in response to chronic stress exposure [87] and decreases in sWAT and vWAT in the obese state [88] in mice. Collectively, these results indicate that aberrant regulation of the genes involved in lipid metabolism may contribute to the pathogenesis of AN in a synergistic manner [84].

In conclusion, heritability can explain a portion of psychological/personality traits in AN, and some known genetic associations are linked to the endocrine disruptions discussed earlier, including genes encoding factors within the HPT, HPG, and HPA axes, with most of these being regulators of appetite and adipocyte lipid metabolism. Additionally, there are alterations in components of monoamine signalling, including dopaminergic and serotonergic pathways, although links to environmental stressor-induced changes in psychology and adipose tissue physiology are unclear. There is also evidence that genetic differences in lipid metabolic pathways underly some cases of AN, in which case aberrant adipocyte lipid metabolism may predispose an individual to stress-induced intensification of the condition.

## Epigenetic modifications in AN and the effect on adipose tissue metabolism

Epigenetic modification refers to potentially heritable alterations in gene expression that occur without changes to the DNA sequence, including methylation, acetylation, phosphorylation, ubiquitination, and SUMOylation of the DNA or histone proteins [89]. There are several aspects of epigenetic regulation of gene expression that underscore its potential importance as the link between chronic stress/environmental factors and the pathogenesis of AN: epigenetic marks can be reversed, are highly dynamic and responsive to external factors, but can also be persistent and modulate long-lasting, reinforcing changes that alter physiology and behaviour. According to the most recent review on epigenetics and eating disorders, thus far, human studies have only assessed DNA methylation status, and exclusively in female patients [90]. Whole blood-based measurements revealed hypomethylation in both adolescent and adult AN females, while buccal cell-based and lymphocyte-based measurements showed unchanged and increased global DNA methylation in adult AN patients, respectively [90]. Despite the association of SNPs in appetite regulatory genes *POMC, CNR1, BDNF, SLC6A4* (encoding serotonin receptor), *DRD4*, and adipose tissue regulatory gene *LEP* (encoding leptin) with AN, methylation status of these genes did not differ between AN and control subjects [90]. Epigenome-wide association studies (EWAS) revealed differentially methylated genes related to lipid metabolism in AN and healthy control subjects [90,91], whereas the lack of follow-up gene expression measurements made it hard to draw convincing conclusions.

Although histone modifications have not been comprehensively studied in AN, histone deacetylase 4 (HDAC4) – an enzyme that facilitates the removal of acetyl groups from histones, is differentially methylated in AN and other eating disorders (reviewed in [92]). Altered methylation status of several 5ʹ-cytosine-phosphate-guanine-3ʹ (CpG) sites in the promoter region of *HDAC4* has been associated with serum oestrogen, fear, learning, appetite and body weight regulation as well as reward processing, etc., which share similar symptomatology with AN [92]. In thigh fat, hypermethylation of *HDAC4* resulted in lowered mRNA expression but higher lipogenic activity after long-term exercise intervention in healthy men [93]. These results collectively suggest a role of HDAC4 in the pathogenesis of AN regarding both appetite regulation and adipose tissue metabolism.

A pilot study revealed that there was hypermethylation of several CpG sites in exon 1 and the MT2 region of the oxytocin receptor gene in the buccal cells of female AN patients compared to healthy controls, and the methylation level was inversely associated with BMI [94]. Circulating oxytocin is lower in AN patients relative to healthy counterparts, which is related to reduced oestrogen – the modulator of oxytocin secretion, as well as its blunted response towards oestrogen stimulation in AN [95]. As reviewed in [95], oxytocin promotes adipogenesis, lipogenesis, as well as lipolysis both *in vitro* and in the WAT of healthy lean rodents *in vivo*, and it also suppresses reward-driven but not hunger-driven food intake, indicating its essential role in maintaining energy homoeostasis. Therefore, hypermethylation of the oxytocin receptor gene may induce unbalanced energy homoeostasis together with other factors such as oestrogen, which eventually contribute to the pathogenesis of AN.

## Early-life stress-induced epigenetic changes in energy metabolism and pathogenesis of AN

As described earlier, there is a high prevalence of AN in adolescent females that may be induced by external triggers, highlighting the importance of understanding how early-life stressors impact physiology later in life. Environmental factors include but are not limited to stress exposure (e.g. psychological, physiological, nutritional, etc.), chemical exposure (e.g. pollutants and drugs/medications), and seasonal/biological rhythm, etc. Epigenetic modification is one major mechanism through which such factors contribute to the pathogenesis of AN. Stress-induced epigenetic changes in energy metabolism have been extensively studied [96–98]. Female singletons whose mothers were exposed to the Dutch famine during gestation had higher circulating total cholesterol, triglycerides and LDL cholesterol than unexposed offspring [97]. A later study reported the association of famine exposure and *LEP* hypermethylation in only the male offspring regardless of the gestational stage of exposure [99]. Similarly, recent research on the offspring of mothers who suffered from the Chinese famine during pregnancy revealed increased total cholesterol [100] and LDL cholesterol [101] levels during adulthood. Moreover, such exposure only increased the risk of dyslipidemia in females, but not males [101], suggesting a sex difference in the susceptibility to abnormal lipid metabolism upon early-life nutritional stress exposure.

As aforementioned, the HPA axis is activated by stimulation of hypothalamic CRF release. In adult mice, long-term social defeat stress exposure, which induces anhedonia, led to decreased DNA methylation in the promoter region of the *Crf* gene and consequently higher mRNA expression in the PVN [102]. Greater gestational CRF is associated with increased risk of foetal growth restriction [103], where the foetuses are smaller in abdominal circumference and lower in abdominal, mid-arm and mid-thigh fat masses compared to the ones that developed without gestational CRF fluctuation [104]. Meanwhile, there is greater adipogenic and lipogenic but lower lipolytic activity in the adipose tissue of those growth-restricted foetuses, accompanied by increased risk of visceral adiposity later in life [105]. In contrast, exposure to 14-day chronic variable mild stress increased methylation in the promoter region of exon 1 and of the intronic sequence between exons 1 and 2 of the *Crf* gene in female adult rats, which was associated with decreased numbers of CRF-immunoreactive neurons in the PVN, whereas in males there were increased *Crf* mRNA-positive cells in the PVN, although both sexes had decreased body weights compared to non-stressed counterparts [106]. Such a sex difference in stress-induced methylation and the stress response was hypothesized to be related to higher basal corticosterone (the form of cortisol in rodents) in the females [106].

Glucocorticoid receptor (GR) activation by cortisol in the hypothalamus and pituitary provide negative feedback to the HPA axis, suppressing the stress response to restore homoeostasis [107]. Maternal stress exposure during pregnancy increased *GR* methylation in the promoter region [108] as well as in exon 1 F [109] of the offspring. This methylation was associated with higher cortisol stimulation under stress [110], which may lead to consistently greater circulating cortisol during chronic stress – consistent with observations in AN patients. Elevated cortisol is associated with the accumulation of vWAT [111] and persistent elevation of corticosterone-impaired non-shivering thermogenesis and promoted lipid storage inside BAT in rats [112]. Indeed, in rats, maternal stress exposure during gestation induced higher baseline corticosterone but lower insulin in the offspring together with defective responses to leptin and ghrelin upon starvation, leading to food intake refractory responses and weight loss [113].

These symptoms are similar to those observed in AN patients, indicating that prenatal stress exposure plays a role in the pathogenesis of AN later in life. Although many of the studies that describe stress-induced changes in hormonal axes and metabolism do not focus on the AN condition, per se, they provide compelling evidence that chronic activation of the HPA axis induces changes that precede metabolic disorders, suggesting that a genetic predisposition to alterations in any of these pathways, might make an individual more susceptible to stress-induced AN. Although there are limitations in modelling this in a clinical setting, there are opportunities to develop animal models that recapitulate some of the features of AN and can be used to further define mechanisms and define molecular targets for pharmacotherapeutics.

## Conclusions and implications for future research

In summary, AN is characterized by a reduction in appetite and adipose tissue mass. Initially, lipid mobilization may increase to restore energy homoeostasis while with prolonged negative energy balance, this is reduced to preserve energy for vital functions to compensate for limited energy intake. Because adipose tissue is a critical endocrine organ that communicates with the rest of the body, including appetite-regulatory regions of the brain, to maintain energy homoeostasis, a reduction in its mass and change in adipocyte function may contribute to the endocrine disruptions that are present in AN, and lead to continued reinforcement of hypophagia and reduced metabolic rate. Traits associated with appetite regulation and lipid metabolism are heritable in AN. Epigenetic modifications may partially explain the differences in gene expression among adipose tissue depots in AN, although the environmentally sensitive nature of these marks makes it difficult to determine whether such modifications are the causes or consequences of AN.

When considering the effects of stress-induced epigenetic modifications on energy homoeostasis, it should be noted that epigenetic modifications are very sensitive to the type and intensity of the stressors, and unlike experimental animals in which there is strict control of housing and diet, the human epigenome may also be affected by diverse factors such as lifestyle [100]. Meanwhile, epigenetic modifications may not necessarily cause changes in gene expression. Thus, human studies with the inclusion of larger sample sizes, and meta-analyses integrating both epigenetic changes and gene/protein expression are expected for future research and the results should be corrected for environmental variables and still be interpreted with due caution. Moreover, although the effect of stress on epigenetic modification of the HPA axis has been comprehensively studied in humans, little is known about the direct effect of stress exposure, especially during early life, on adipose tissue metabolism and sequential effects on the endocrine system in AN. This is in part due to the limited amount of adipose tissue in AN patients, which is of vital importance for life maintenance. Although such studies have been carried out with rodents, focusing on maternal distress, whether the effects are conserved across species, both in mammals and non-mammals, is important for the understanding of adaptative mechanisms from an evolutionary perspective. Because AN patients display depot-specific changes in WAT mass and BAT activation, the specification of effects and corresponding mechanism in each adipose tissue depot are needed in future studies. Thus, animal models should be developed and adopted to explore the effects of early-life stress exposure on both early and later life adipose tissue physiology to further elucidate the mechanisms involved, which will shed light on novel therapeutic targets in stress-induced metabolic abnormalities.
